# Status of 10 targeted genes of non‐small cell lung cancer in eastern China: A study of 884 patients based on NGS in a single institution

**DOI:** 10.1111/1759-7714.13577

**Published:** 2020-07-30

**Authors:** Dan Li, Li Ding, Wenwen Ran, Yan Huang, Guangqi Li, Chengqin Wang, Yujing Xiao, Xiaonan Wang, Dongliang Lin, Xiaoming Xing

**Affiliations:** ^1^ Department of Pathology The Affiliated Hospital of Qingdao University Qingdao China; ^2^ Medical Affairs Department The Affiliated Hospital of Qingdao University Qingdao China; ^3^ Department of Human Resources The Affiliated Hospital of Qingdao University Qingdao China

**Keywords:** Clinicopathological characteristics, gene mutation, next‐generation sequencing, non‐small cell lung cancer

## Abstract

**Background:**

The status of targeted genes and the association between targeted genes and clinicopathological features in Chinese lung cancer patients remains to be elucidated.

**Methods:**

The status of 10 targeted genes was evaluated by next‐generation sequencing (NGS) in 884 non‐small cell lung cancer (NSCLC) patients. The relationship between gene alterations and clinicopathological characters was analyzed.

**Results:**

Overall, 684 (77.4%) patients harbored gene alterations, and *EGFR* (510, 57.7%) was found to be the most common type of mutation followed by *KRAS* (91, 10.3%), *HER2* (38, 4.3%), *PIK3CA* (32, 3.6%), *ALK* (21, 2.4%), *BRAF* (10, 1.1%), *ROS1* (5, 0.6%), *RET* (5, 0.6%), *MET* (4, 0.5%) and *NRAS* (1, 0.1%)*.* Gene alterations were more frequent in females, non‐smokers and adenocarcinoma (*P* < 0.001). *EGFR* mutations were associated with women, non‐smokers, normal level of serum tumor markers, and adenocarcinoma *(P* < 0.001). Patients without lymph node metastasis (*P* = 0.012), or early stage disease (*P* < 0.001) exhibited a higher *EGFR* mutation rate. *KRAS* mutations tended to arise in men (*P* < 0.001), smokers (*P* < 0.001) and patients with higher levels of serum tumor markers (*P* = 0.048). A mucus‐producing component was associated with *KRAS* (*P* < 0.001), *ROS1* (*P* = 0.033) and *ALK* (*P* < 0.001) alterations. *ALK* and *ROS1* rearrangements were more frequent in micropapillary structures (*P* = 0.004, *P* = 0.012). *BRAF* mutation was associated with advanced disease patients and micropapillary structure (*P* < 0.001). *PIK3CA* mutation was more likely to be found in elderly patients (*P* = 0.014). Some patients had synchronous gene alterations, including *EGFR/PIK3CA*, *EGFR/HER2*, *HER2/KRAS*, *EGFR/KRAS*, *EGFR/ROS1*, *EGFR/NRAS*, *KRAS/PIK3CA*, *KRAS/PIK3CA/HER2.*

**Conclusions:**

Most patients had at least one genetic alteration, and individual patients harbored synchronous mutation. Each gene alteration had unique clinicopathological characteristics.

**Key points:**

**Significant findings of the study:**

This study revealed the frequency and distribution of 10 targeted gene abnormalities and their association with clinicopathological parameters of Chinese non‐small cell lung cancer (NSCLC) patients in eastern China.

**What this study adds:**

Some rare synchronous mutations were detected in our study by next‐generation sequencing (NGS).

## Graphical abstract

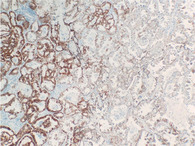

In this research, we investigated the frequency of common driving genes in 884 patients with non‐small cell lung cancer and analyzed their association with clinicopathologic parameters in in eastern China. Most patients had at least one genetic alteration, and individual patients harbored synchronous mutation. Each gene alteration has unique clinicopathologic characteristics.

## Introduction

Lung cancer is a common malignant tumor and causes the largest number of cancer‐related deaths worldwide.^1^, ^2^ Non‐small cell lung cancer (NSCLC) is the most common histologic type of lung cancer, accounting for approximately 80% of all cases of lung cancer. NSCLC mainly includes adenocarcinoma, adenosquamous carcinoma, squamous cell carcinoma and large cell carcinoma.^3^ In recent years, oncogene aberration has been widely studied in various cancers, especially in lung carcinoma.^4^, ^5^ New and emerging molecular targeted therapies have achieved substantial progress in NSCLC due to understanding the molecular origins, signaling transduction pathway and metabolic process in lung cancer. Research into the role of tyrosine kinase inhibitors in *EGFR* gene mutations has greatly improved the prognosis of lung cancer patients with *EGFR* activating mutation^6^, ^7^ and with the development of molecular targeted drug research, except for *EGFR* mutation, many other types of targeted gene aberrations have been identified, including *HER2*,^8^ *BRAF*,^9^ *KRAS* mutations,^10^ and *ALK* rearrangement.^11^ Targeted drugs can provide optimal efficacy and prolong survival time in activating mutation NSCLC patients.^12^

There are many methods for gene detection for targeted drugs. Compared with traditional methods, next‐generation sequencing (NGS) reduces the cost of testing and can comprehensively screen different gene alterations to provide a reliable and effective therapeutic schedule for individualized treatment. Here, we examined the status of *EGFR*, *HER2*, *BRAF*, *KRAS*, *ALK*, *MET*, *NRAS*, *PIK3CA*, *RET*, and *ROS1* in 884 patients with NSCLC who had undergone surgical resection, bronchoscopy biopsy or percutaneous transthoracic biopsy by NGS in our institution. We attempted to investigate the frequency and distribution of 10 targeted gene abnormalities and analyze their association with clinicopathological parameters.

## Methods

### Patients

This study included all NSCLC patients who underwent surgical resection (714/884) and bronchoscopy biopsy and percutaneous transthoracic biopsy (170/884) from June 2017 to September 2018 in the Affiliated Hospital of Qingdao University. A histological diagnosis had been sufficiently confirmed by two experienced pulmonary pathologists. A total of 884 patients (486 female and 398 males) were included in the present study. Bronchoscopy and puncture biopsy specimens from patients diagnosed with NSCLC‐NOS were not included in this study. This study was approved by the Ethics Committee of the Affiliated Hospital of Qingdao University (No. QYFY WZLL 25784). Written informed consent was obtained from all patients.

### Clinicopathological characteristics

Histologic subtypes were classified according to the 2015 WHO histologic classification of lung cancer.^3^ All patients were staged according to the eighth edition of the TNM classification of the American Joint Committee on Cancer (AJCC) for lung cancer.^13^ We collected clinicopathological data from patients' electronic medical record database, including age, gender, smoking history, tumor location, metastasis, pathological TNM stage and the level of serum tumor markers associated with lung cancer (including CEA, CK19, CA125, SCCA, ProGRP and NSE).

### Methods of DNA extraction and next‐generation sequencing

All tumor tissue samples were fixed in 10% neutral formalin solution, embedded in paraffin, and 3 μM sections were prepared for hematoxylin and eosin (H&E). There were enough tumor cells for gene detection. We used QIAamp DNA formalin‐fixed paraffin‐embedded (FFPE) tissue kit (Qiagen, Valencia, CA, Germany) to extract genomic DNA from FFPE tissues. The concentration of genomic DNA was detected by the Qubit dsDNA Assay Kit (Invitrogen, Carlsbad, CA, USA). We used human *EGFR/KRAS/BRAF/PIK3CA/ALK/ROS1/HER2/MET/RET/NRAS* mutation test kit (Geneis, Beijing, China) to prepare the libraries. PCR products were enriched, purified, concentrated and a gene probe pool was used to capture the libraries. A quantity and quality inspection was performed using the Bioanalyzer 2100 system (Agilent Technologies, Santa Clara, USA). Under the condition that the concentration satisfied the requirement, the NGS was conducted on an Illumina MiniSeq system (Illumina, San Diego, USA), utilizing the MiniSeq High Output Reagent Kit (300 cycles) (Illumina) to sequence the genomic DNA with 15–18 pooled libraries, and paired‐end with 2 × 151 bp. We split the original sequencing records to determine whether the sample data was qualified, and removed the inferior quality dates. Alterations were recognized by BWA, Freebayes and Annoval. We used Crest, Factera, ionCOPY, FreeBayes and Annoval to analyze and annotate the mutation information of binary sequence alignment/map files. In the process of verifying the NGS analysis, the background noise cutoff value for the single nucleotide variation was selected as 1%. In this research, the minimum requirement for sequencing depth was 500x. All procedures were conducted following the manufacturers' protocols.

### Immunohistochemistry

Immunohistochemical analysis was performed on PPFE sections using the HER2 (Ventana Medical Systems, Tucson, AZ, USA) antibody. HER2 staining was performed on a VENTANA Benchmark XT automated system (Ventana Medical Systems, Inc., Tucson, AZ, USA).

### Statistical analysis

The statistical analysis of clinicopathological features was carried out by Chi‐square test or Fisher's exact test to explore the association between 10 targeted gene alterations frequency and clinicopathological profiles. All statistical analyses were evaluated using the SPSS 24.0 software (SPSS, Chicago, IL). A *P* < 0.05 was considered statistically significant.

## Results

### Patient characteristics

Detailed clinicopathological information is summarized in Table 1. In this study, 868 patients had not been treated with preoperative chemotherapy or targeted therapy, 10 patients had received targeted treatment, and six patients had received preoperative adjuvant chemo‐radiotherapy. There were 486 (55.0%) females and 398 (45.0%) males, and 266 (30.1%) patients were current or former smokers. The average age was 60.1 years (range, 22–87 years). The pathological analysis identified that 92.1% (814/884) of the samples were from adenocarcinoma, 6.1% (54/884) from squamous cell carcinoma, 1.0% (9/884) from adenosquamous carcinoma and 0.8% (7/884) from large cell carcinoma.

**Table 1:** Clinicopathological profiles of 884 patients

Clinicopathological profiles	No. of patients (%)
Sex	
Male	398 (45.0)
Female	486 (55.0)
Age	
Median	61.0
Average	60.1
Range	22–87
Smoking history	
Non‐smoker	618 (69.9)
Former/current smoker	266 (30.1)
Serum tumor markers	
Normal	357 (40.4)
Abnormal	527 (59.6)
Tumor location	
Left lung	394 (44.5)
Right lung	488 (55.3)
Histologic subtype	
AIS	5 (0.6)
MIA	58 (6.6)
Invasive adenocarcinoma	751 (85.9)
Lepidic	123 (13.9)
Acinar	384 (43.4)
Papillary	89 (12.2)
Solid	56 (10.1)
Micropapillary	7 (0.8)
IMA	20 (2.3)
SCC	54 (6.1)
Adenosquamous carcinoma	9 (1.0)
LCC	7 (0.8)
TNM stage	
I	474(53.6)
II	95 (10.7)
III	184 (20.8)
IV	131 (14.8)

AIS, adenocarcinoma in situ; IMA, invasive mucinous adenocarcinoma; LCC, large cell carcinoma; MIA, minimally invasive adenocarcinoma; SCC, squamous cell carcinoma.

Table 2 shows the comparison of the mutation rate of 10 targeted genes in different genders, age groups, smoking history and histologic types. The mutation rate in females was 86.0% (418/486), which was significantly higher than that in males (66.8%, 266/398) (*P* < 0.001). There was no statistical difference between the patients over 60 years old (78.5%, 351/447) and those under the age of 60 (76.2%, 333/437) (*P* = 0.409). Gene mutations were detected in 84.1% (520/618) non‐smokers, which was significantly higher than that in former or current smokers (61.7%, 164/266) (*P* < 0.001). Overall, 81.7% (672/823) of samples with an adenocarcinoma component, including adenocarcinoma and adenosquamous carcinoma, had gene mutations. In contrast, only 19.7% (12/61) of samples without an adenocarcinoma component, including squamous cell carcinoma and large cell carcinoma, had gene mutations (*P* < 0.001).

**Table 2:** Comparison of the mutation rate of 10 targeted genes in different genders, age groups, smoking history and histologic types

Group	No (n)	Mutant (%)	Wild‐type (%)	χ2	*P*‐value
Gender (P)				45.953	**<0.001**
Male	398	266 (66.8)	132 (33.2)		
Female	486	418 (86.0)	68 (14.0)		
Age (P)				0.681	0.409
<60	437	333 (76.2)	104 (23.8)		
≥60	447	351 (78.5)	96 (21.5)		
Smoking history (P)				53.721	**<0.001**
Non‐smokers	618	520 (84.1)	98 (15.9)		
Former/current smokers	266	164 (61.7)	102 (38.3)		
Histologic types (P)				124.625	**<0.001**
With adenocarcinoma component	823	672 (81.7)	151 (18.3)		
Without adenocarcinoma component	61	12 (19.7)	49 (80.3)		

P, P refers to the comparison with different genders, age groups, smoking history and histologic types. With adenocarcinoma component, includes adenocarcinoma and adenosquamous carcinoma; without adenocarcinoma component, includes squamous cell carcinoma and large cell carcinoma.

### Frequency of 10 targeted gene alterations

In this study, the mean depth of sequencing was 1000×. Of 884 patients, 684 (77.4%) patients had at least one gene alteration, and 33 (3.7%) samples harbored more than one driver gene alteration. The *EGFR*, *KRAS*, *HER2*, *PIK3CA*, *ALK*, *BRAF, ROS1*, *RET*, *MET*, and *NRAS* gene alteration rates were 57.7% (510/884), 10.3% (91/884), 4.3% (38/884), 3.6% (32/884), 2.4% (21/884), 1.1% (10/884), 0.6% (5/884), 0.6% (5/884), 0.5% (4/884) and 0.1% (1/884), respectively. Moreover, 33 (3.7%) patients had another synchronous gene alteration, *EGFR/PIK3CA* (19, 2.1%) was the most frequent coalteration, followed by *EGFR/HER2* (6, 0.7%), *HER2/KRAS* (3, 0.3%), *EGFR/KRAS* (1, 0.1%), *EGFR/ROS1* (1, 0.1%), *EGFR/NRAS* (1, 0.1%), *KRAS/PIK3CA* (1, 0.1%). Only one case harbored a triple *KRAS/PIK3CA/HER2* coalteration (0.1%, 1/884). Detailed information of multiple alteration combinations can be found in Table S1. Multiple alteration *EGFR* exon 21 L858+*PIK3CA* exon 9 E545K showed the highest incidence rate (15.2%, 5/33) followed by *EGFR* exon 19 del +*PIK3CA* exon 20 H1047R (12.1%, 4/33). Specific mutation frequency of each gene in surgical specimens and biopsy specimens is shown in Table S2. In general, surgical specimens were from patients in early stage disease, and puncture or bronchoscopic biopsy specimens were from patients in advanced stage disease. *EGFR* mutation occurred mostly in patients with early stage disease (*P* < 0.001), whereas *KRAS* and *HER2* alterations more frequently occurred in patients in advanced stage disease (*P* = 0.008, *P* = 0.005, respectively).

### *EGFR* mutation status and association with clinicopathological features

In this study, 510 (57.7%) patients harbored *EGFR* mutation, 28 (5.5%) specimens possessed another synchronous gene alteration, *EGFR/PIK3CA* (19/28) was the most frequent coalteration, followed by *EGFR/HER2* (6/28). In addition, double mutations involving the same or different exons in *EGFR* were observed in 29 patients, accounting for 3.3% (29/884) of all patients. The detailed information of *EGFR* mutations with clinicopathological characteristics of NSCLC is shown in Table 3. Overall, 31.4% (278/884) of patients had an exon 21 L858R mutation, followed by exon 19 del in 20.8% (184/884) patients. The other four mutation subtypes higher than 1.0% were exon 18 G719X, exon 20 T790M, exon 21 L861Q and exon 20 S768I, and the frequencies were 2.9% (26/884), 1.6% (14/884), 1.5% (13/884), and 1.2% (11/884), respectively. Furthermore, five rare mutations were detected in our study, and the specific mutation phenotype is as follows: exon 18 p.Leu707Trp, exon 18 p.Glu709_Thr710delinsAsp, exon 20 p.Gly796Ser, exon 19 p.Glu749_Ser752delinsAsp, exon 20 p.His773_Val774delinsArgMet. Of the 14 cases with T790M mutation, five cases were accompanied by 19 del mutation, and nine cases were accompanied by 21 L858R mutation. Seven of the patients with 20 T790M mutation had an EGFR‐TKI treatment history.

**Table 3:** Associations of *EGFR* mutation with clinicopathological characteristics of NSCLC

	No. of patients							
Clinicopathological characteristics	No	Total *EGFR mutation*	18 G719X	19 del	20 S768I	20 T790M	21 L858R	21 L861Q
No		510	26	184	11	14	278	13
Age (P)		0.575	0.846	0.995	0.120	0.116	0.352	0.812
<60	437	248	14	91	8	4	131	6
≥60	447	262	12	93	3	10	147	7
Sex(P)		**<0.001**	0.906	**<0.001**	0.738	0.480	**<0.001**	0.632
Male	398	157	12	56	6	5	80	5
Female	486	353	14	128	5	9	198	8
Serum tumor markers (P)		**<0.001**	0.543	**0.032**	**0.033**	**0.045**	**0.004**	0.669
Normal	357	240	9	87	1	2	132	6
Abnormal	527	270	17	97	10	12	146	7
Gross type (P)		**<0.001**	0.680	**0.044**	0.328	0.696	**0.032**	0.584
Central type	70	24	1	6	0	2	14	0
Peripheral type	814	486	25	176	11	12	264	13
Smoking history (P)		**<0.001**	0.939	**<0.001**	0.900	0.111	**0.020**	0.802
Non‐smokers	618	431	18	156	7	13	238	10
Former/current smokers	266	79	8	28	4	1	40	3
Histology (P*)		**<0.001**	0.309	**<0.001**	0.756	0.620	**<0.001**	0.932
With adenocarcinoma component	823	509	26	184	11	14	277	13
AIS	5	2	0	1	0	0	1	0
MIA	58	21	0	6	0	0	15	0
Lepidic	123	68	1	17	0	0	47	1
Acinar	384	278	15	100	7	6	153	9
Papillary	89	71	6	31	0	1	31	1
Solid	56	12	1	3	1	0	7	0
Micropapillary	7	4	0	2	0	0	2	0
IMA	20	1	0	0	0	0	0	0
Adenosquamous carcinoma	9	6	0	2	0	0	4	0
Without adenocarcinoma component	61	1	0	0	0	0	1	0
SCC	54	1	0	0	0	0	1	0
LCC	7	0	0	0	0	0	0	0
TNM stage (P)		**<0.001**	0.599	0.336	1.000	0.158	**0.001**	1.000
I + II	569	358	18	124	7	6	201	8
III + IV	315	152	8	60	4	8	77	5

With adenocarcinoma component, includes adenocarcinoma and adenosquamous carcinoma; Without adenocarcinoma component, includes squamous cell carcinoma and large cell carcinoma; P, P refers to the comparison with different age groups, genders, serum tumor markers, gross type, smoking history and TNM stage; P*, P* refers to the comparison between with adenocarcinoma component and without adenocarcinoma component.

AIS, adenocarcinoma in situ; IMA, invasive mucinous adenocarcinoma; LCC, large cell carcinoma; MIA, minimally invasive adenocarcinoma; SCC, squamous cell carcinoma.

As shown in Table 3, the *EGFR* mutation was significantly associated with women, non‐smoker, and normal serum tumor markers level (both *P* < 0.001). The patients without lymph node metastasis (*P* = 0.012) in early stage disease (*P* < 0.001) exhibited a higher *EGFR* mutation rate. Patients with adenocarcinoma (*P* < 0.001) and peripheral tumors (*P* < 0.001) were found to be more likely to have *EGFR* mutations.

### *ALK* rearrangement status and association with clinicopathological features

A total of 21 patients (2.4%) with invasive adenocarcinoma had *ALK* rearrangement, and all demonstrated echinoderm microtubule associated protein‐like 4 (EML4) rearrangement. As shown in Table 4, *ALK* rearrangement was more frequent in adenocarcinomas with mucus‐producing component (*P* < 0.001), micropapillary structure (*P* = 0.004) and invasion of the visceral pleura of the lung (*P* = 0.048). No difference was detected in age, gender, smoking history, tumor location and TNM stage between patients with and without *ALK* rearrangement (*P* > 0.05).

**Table 4:** Nine targeted gene alterations status and association with histological characteristics of NSCLC

	No. of patients									
Histology	No	*ALK*	*ROS1*	*BRAF*	*KRAS*	*HER2*	*PIK3CA*	*RET*	*MET*	*NRAS*
With or without adenocarcinoma component (P*)		0.207	0.699	1.000	0.062	0.684	**0.001**	0.542	0.715	1.000
With adenocarcinoma component	823	21	5	9	89	36	25	5	4	1
AIS	5	0	0	0	1	0	0	0	0	0
MIA	58	0	0	0	5	10	0	0	1	0
Lepidic	123	1	0	0	11	11	1	0	2	0
Acinar	384	6	2	1	27	8	14	5	1	1
Papillary	89	3	2	1	6	2	3	0	0	0
Solid	56	5	0	0	11	1	1	0	0	0
Micropapillary	7	1	0	1	0	0	0	0	0	0
IMA	20	3	0	1	10	1	0	0	0	0
Adenosquamous carcinoma	9	0	0	0	0	2	0	0	0	0
Without adenocarcinoma component	61	0	0	1	2	2	7	0	0	0
SCC	54	0	0	1	2	2	7	0	0	0
LCC	7	0	0	0	0	0	0	0	0	0
Mucus‐producing component (P)		**<0.001**	**0.033**	0.440	**<0.001**	0.643	0.848	1.000	1.000	1.000
Present	57	8	2	1	17	3	1	0	0	0
Absent	664	11	2	6	49	21	21	4	3	1
Micropapillary structure (P)		**0.004**	**0.012**	**<0.001**	0.375	0.462	0.618	0.226	0.542	1.000
Present	164	10	3	6	12	4	6	2	1	0
Absent	553	9	0	0	53	20	16	2	2	1
Invasion of visceral pleura (P)		**0.048**	0.511	0.217	0.130	0.395	0.132	1.000	1.000	1.000
Present	152	8	1	3	9	7	8	1	0	0
Absent	566	11	2	3	56	18	14	3	3	1

With adenocarcinoma component, includes adenocarcinoma and adenosquamous carcinoma; without adenocarcinoma component, includes squamous cell carcinoma and large cell carcinoma; P*. P* refers to the comparison between with adenocarcinoma component and without adenocarcinoma component. P, P refers to the comparison of mucus‐producing component, micropapillary structure, and visceral pleura invasion.

AIS, adenocarcinoma in situ; IMA, invasive mucinous adenocarcinoma; LCC, large cell carcinoma; MIA, minimally invasive adenocarcinoma; SCC, squamous cell carcinoma.

### *ROS1* fusion status and association with clinicopathological features

*ROS1* fusion was detected in five patients with adenocarcinoma. The patterns were *CD74‐ROS1* (three cases), *SDC4‐ROS1* (one case) and *GOPC‐ROS1* (one case), respectively. The patient with *GOPC‐ROS1* fusion also carried a synchronous *EGFR* 19 del mutation. As shown in Table 4, *ROS1* rearrangement was more frequent in adenocarcinomas with mucus‐producing component (*P* = 0.033) and micropapillary structure (*P* = 0.012). No correlation was detected between *ROS1* expression and other clinicopathological characteristics (*P* > 0.05).

### *BRAF* mutation status and association with clinicopathological features

*BRAF* mutation was detected in 10 (1.1%) cases. Of the 10 cases, there were nine invasive adenocarcinomas and one squamous cell carcinoma. Seven cases demonstrated *BRAF* V600E mutation in exon 15, and three cases demonstrated *BRAF* G469A mutation in exon 11. As shown in Tables 4 and 5, nine patients with *BRAF* mutation had advanced stage III or IV NSCLC . *BRAF* mutation was significantly associated with advanced disease patients (*P* < 0.001) and micropapillary structure (*P* < 0.001).

**Table 5:** Nine targeted gene alteration status and association with age, gender, serum tumor markers, tumor location, smoking history, and TNM stage of NSCLC

	No. of patients									
Clinicopathological characteristics	No	*ALK*	*ROS1*	*BRAF*	*KRAS*	*HER2*	*PIK3CA*	*RET*	*MET*	*NRAS*
Age (P)		0.247	0.356	0.723	0.509	0.162	**0.014**	0.383	0.632	0.506
<60	437	13	4	6	42	23	9	1	1	0
≥60	447	8	1	4	49	15	23	4	3	1
Sex (P)		0.276	1.000	0.202	**<0.001**	0.300	0.193	1.000	0.481	1.000
Male	398	7	2	7	70	14	18	2	3	0
Female	486	14	3	3	21	24	14	3	1	1
Serum tumor markers (P)		0.257	0.635	1.000	**0.048**	0.907	0.735	0.075	0.906	0.404
Normal	357	11	1	4	28	15	12	0	1	1
Abnormal	353	10	4	6	63	23	20	5	3	0
Gross type (P)		0.783	0.661	0.404	0.189	0.221	**<0.001**	0.316	0.719	1.000
Central type	70	2	0	2	4	5	8	1	0	0
Peripheral type	814	19	5	8	87	33	24	4	4	1
Smoking history (P)		0.878	0.996	0.301	**<0.001**	0.604	0.590	1.000	0.322	1.000
Non‐smokers	618	15	4	5	35	28	21	3	4	1
Former/current smokers	266	6	1	5	56	10	11	2	0	0
Site of tumor location (P)		0.873	0.511	0.191	0.144	0.755	0.412	0.806	0.776	1.000
Left lung	394	9	1	7	34	16	12	3	1	0
Right lung	488	12	4	3	57	22	20	2	3	1
TNM stage (P)		0.105	0.501	**0.001**	0.291	0.394	0.176	1.000	1.000	1.000
I + II	596	10	2	1	54	22	17	3	3	1
III + IV	315	11	3	9	37	16	15	2	1	0

P, P refers to the comparison with different age groups, genders, serum tumor markers, smoking history, site of tumor location, and TNM stage.

### *KRAS*, *PIK3CA*, *HER2*, *MET*, *RET* and *NRAS* gene alterations status and association with clinicopathological features

As shown in Tables 4 and 5, *KRAS* mutations were found more frequently in men (*P* < 0.001), former or current smokers (*P* < 0.001), and patients who had a higher level of serum tumor markers (*P* = 0.048). Moreover, *KRAS* mutations were correlated with the mucus‐producing component (*P* < 0.001). *PIK3CA* mutation was significantly more frequent in elderly patients (*P* = 0.014) with central invasive adenocarcinoma (*P* < 0.001). Among the 32 samples with *PIK3CA* mutation, 19 samples harbored concurrent *EGFR* mutations, and two samples had *KRAS* mutations. *HER2* gene alterations were detected in 38 cases including gene amplification (19 cases) and mutation located in exon 20 (19 cases). In histological type, 94.7% (36/38) *HER2* alterations were adenocarcinoma. We failed to find any significant association between amplification or point mutation with clinicopathological parameters. Synchronous *EGFR* mutation or *KRAS* mutation was detected in 10 patients harboring *HER2* amplification. *RET* fusion was found in five patients, three cases had *KIF5B‐RET* fusion, and two cases *CCDC6‐RET* fusion. *MET* gene alteration was observed in four patients, including two cases with gene amplification and two cases with exon 14 skipping mutation. No significant association was observed in any clinicopathological features of *HER2* alteration (*P* > 0.05). The number of samples with rare mutations such as *MET*, *RET* and *NRAS* was too low for statistical analysis.

## Discussion

In this study, we investigated the relationship between 10 targeted gene alterations (including *EGFR*, *HER2*, *BRAF*, *KRAS*, *ALK*, *MET*, *NRAS*, *PIK3CA*, *RET*, and *ROS1*) and the clinicopathological features in 884 patients with NSCLC. Our data showed that approximately 75% of patients had at least one gene alteration. The most popular mutation was *EGFR* which was detected in more than half of the patients (57.7%), followed by *KRAS* (10.3%), *HER2* (4.3%), *PIK3CA* (3.6%), *ALK* (2.4%), *BRAF* (1.1%). The other four targeted gene alteration rates were less than 1%.

To the best of our knowledge, somatic mutations of *EGFR* are more common in Asian patients.^14^ In our study, the frequency of *EGFR* mutations was higher than previous reports in Chinese NSCLC cohorts,^15^, ^16^ but was similar to recent studies.^17^, ^18^ This may be due to an improvement in medical level and detection methods, whereby more patients are diagnosed in early stage disease which improves the positive detection rate. The higher proportion of adenocarcinoma patients in this study is also a possible cause. *EGFR* mutation is more likely to be detected in women, non‐smokers, and early stage patients without lymph node and organ metastasis, as previously reported.^19^, ^20^ In our study, the most common treatment‐sensitive activating mutations were *EGFR* L858R and 19 del mutations, followed by G719 X and L861Q, which was consistent with previous reports in the literature.^15^, ^18^ Gefitinib and erlotinib have proved significantly curative effectiveness in the treatment of sensitive *EGFR* mutation.^21^ The 20 T790M mutation is the most common secondary resistance mechanism in *EGFR* and is associated with poorer survival rates in NSCLC.^22^ Therefore, the detection of *EGFR* gene mutation status is a prerequisite for targeted drugs to be administered to patients. There were seven patients who had a T790M secondary mutation in our study. It is worth noting that we also detected primary T790M mutation in seven patients (0.8%) without any history of TKI therapy. All seven patients had other *EGFR* activating mutations. Primary T790M mutation has been reported to coexist more commonly with L858R, whereas acquired mutation more likely coexists with 19 del,^23^, ^24^ indicating different mechanisms are present between the two. Previous studies have shown that whilst patients with primary or acquired T790M mutation show significant differences in certain clinical features, and may benefit from osimertinib therapy, those patients with acquired T790M mutation may have a higher overall survival rate than those with primary T790M mutation.^23^, ^24^ This may be due to the fact that 19 del could destroy the inactive conformation of the *EGFR* kinase domain and enhance EGFR‐TKI sensitivity. Searching the databases and reviewing previous studies, the clinical significance of most rare mutations is unclear, and some mutations may possibly be related to TKI resistance.^25^

The fusion of *ALK*, *ROS1* and *MET* are rare genetic alterations in patients with NSCLC who can benefit from crizotinib or vandetanib therapy.^26^, ^27^ Similarly, these three kinds of fusion occur easily, predominantly in younger non‐smokers, and in lung adenocarcinoma patients.^28^, ^29^ *ALK* gene rearrangement has been reported to be present in about 3%–8% of NSCLC patients,^4^, ^30^ and *ROS1* and *RET* fusion reported to be present in about 1%–2% of patients with NSCLC.^11^ In our study, the rate of occurrence of *ALK*, *ROS1* and *RET* fusion was slightly lower. This may be due to regional or demographic differences. A retrospective analysis by Zhao *et al*.^31^ showed that age, gender, specimen type, histologic type, and smoking history were correlated with *ALK* status. However, in our study, apart from histologic type, no significant association between *ALK* status and clinicopathological features was observed. This difference may result from different sample size or geographic differences. *ALK* rearrangement was more frequent in adenocarcinomas with a mucus‐producing component, micropapillary structure and visceral pleura invasion. This may be the reason for the poor prognosis in patients with *ALK* rearrangement. Zhao *et al*. also reported that *ALK* rearrangement was more common in patients with invasive mucinous adenocarcinoma and solid‐predominant invasive adenocarcinoma.^31^ Our data showed that *ALK*, *ROS1*, *BRAF* or *KRAS* gene alterations were significantly more frequent in adenocarcinomas with a mucus‐producing component or micropapillary component, and these two components could be a nonpredominant component in some cases. This suggests that attention should be paid not only to the predominant component but also to other nonpredominant components in NSCLC. Pathologists should list every histologic component more than 5% in pathological reports as suggested by WHO histologic classification.^3^ *ALK*, *ROS1* or *RET* fusion was not detected in squamous cell carcinomas and large cell carcinomas in our study. The result suggested that these fusions may be very rare in squamous cell carcinomas and large cell carcinomas.^32^

The study by Lin *et al*. revealed that the *BRAF* mutation rate in Chinese NSCLC patients was 2.8%, and that V600 and G469 were the two most common mutation types.^33^ *BRAF* mutation rate is higher in patients with advanced lung adenocarcinoma. Only V600 and G469 mutations were found in our study, and the mutation rate was only 1.1%. Most of the patients in our study were in early‐stage disease, and therefore the *BRAF* mutation rate was lower in our study.

**Figure 1:**
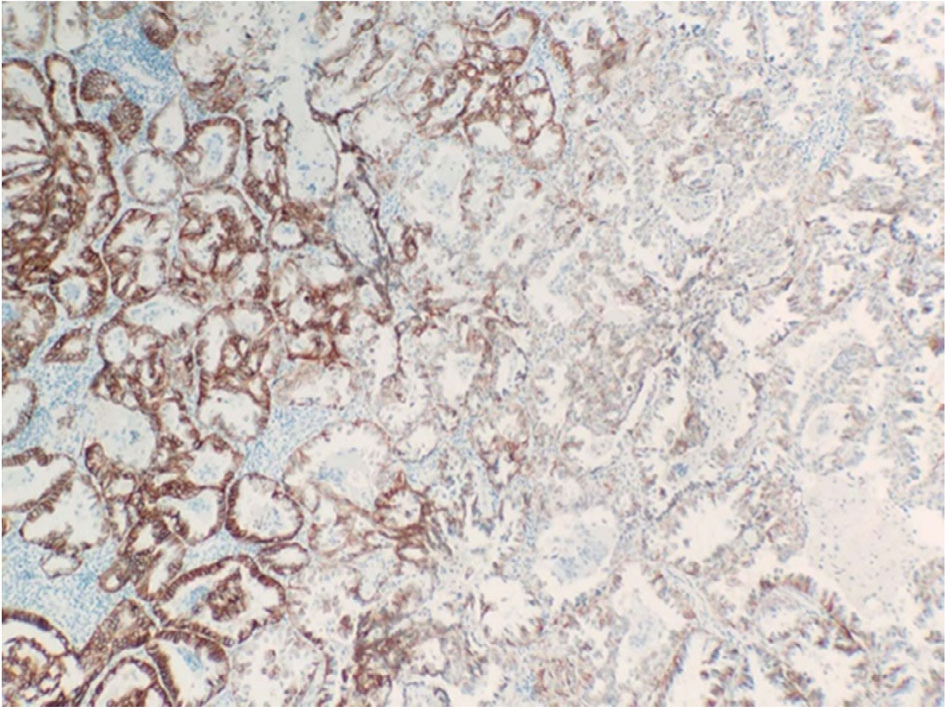
Immunohistochemistry for HER2 protein expression (IHC ×100).

*HER2* alteration has been reported to be more common in women, non‐smokers, Asians and patients with adenocarcinomas, which is similar to those with *EGFR* mutation.^9^, ^34^, ^35^ In our patient cohort, we found that *HER2* alteration was more frequently observed in adenocarcinoma than other histological types, whereas there was no significant association of *HER2* amplification or mutation with gender, age and smoking status in our research, and another study supported our views.^36^ Ken *et al*.^37^ reported that *HER2* amplification may be a possible mechanism for acquired resistance to EGFR‐TKI without the *EGFR* T790M mutation. In this study, there was a patient carrying an *EGFR* 19 del mutation accompanied by primary *HER2* amplification, which showed a response to EGFR‐TKI treatment. This case suggested that primary *HER2* amplification was not indicative of EGFR‐TKI resistance. Immunohistochemistry for HER2 was performed on the tumor tissue in this case, and immunohistochemical stain showed that the expression of HER2 protein had significant heterogeneity (some of the tumor cells were negative for HER2) (Fig 1). Intratumoral heterogeneity therefore explains the effectiveness of EGFR‐TKI therapy in this patient.

A previous study showed that targetable activating alterations in lung cancer genes, such as *EGFR*, *ALK*, *RET* and *ROS1*, are mutually exclusive molecular events.^30^ Recently, however, some cases of NSCLC with coalteration have been reported in the literature,^38^, ^39^, ^40^, ^41^ and these coaltered patients may benefit from treatment with more than one targeted drug.^42^ There were 33 cases (3.7%) with coalteration in our study, including *EGFR/PIK3CA* (2.1%, 19 cases), *EGFR/HER2* (0.7%, six cases), *HER2/KRAS* (0.3%, three cases), *EGFR/KRAS* (0.1%, one case), *EGFR/ROS1* (0.1%, one case), *EGFR/NRAS* (0.1%, one case), *KRAS/PIK3CA* (0.1%, one case), and a triple *KRAS/PIK3CA/HER2* (0.1%, one case) coalteration. Previous studies revealed that *KRAS*, *BRAF*, and *PIK3CA* mutation have been related to efficacy of EGFR‐TKI, metastasis or overall survival.^43^ *PIK3CA* mutations frequently coexist with *EGFR* or *KRAS* mutations. Evidence has suggested that the combination of *KRAS* and *EGFR* mutation may have a negative impact on the efficacy of TKI treatment,^41^ but EGFR‐TKI may be an effective choice for the treatment of patients with NSCLC with comutation of *EGFR/KRAS*.^44^ Zeng *et al*.^45^reported a case of adenocarcinoma having acquired *GOPC‐ROS1* rearrangement after treatment with osimertinib. These authors suggested that *GOPC‐ROS1* rearrangement was a novel acquired resistance mechanism to osimertinib. In our research, a comutation of *EGFR* 19 del with *GOPC‐ROS1* rearrangement was detected in a 60‐year‐old smoking male with an adenocarcinoma. The patient did not undergo anti‐EGFR therapy before a genetic test, indicating it was a coexisting primary *EGFR* exon 19 del plus *GOPC‐ROS1* rearrangement rather than an acquired one. The patient did not undergo targeted therapy after surgery, so we cannot evaluate the efficiency of drug targeted therapy. Moreover, we encountered a unique patient harboring a triple *KRAS/PIK3CA/HER2* alteration. The patient was a 60‐year‐old non‐smoking female with an invasive adenocarcinoma (T1bN1M0). With more and more coalterations being found in NSCLC patients, we need to investigate the mechanism of their occurrence, because the efficiency of relevant targeted drugs is unclear in these patients.

In conclusion, our research revealed the status of 10 targeted genes of 884 NSCLCs in eastern China. Most of the patients had at least one gene alteration, and our study confirmed that NGS is a reliable and effective method for gene detection in NSCLCs.

## Disclosure

No authors report any conflict of interest.

## Supporting information


**Table S1** Supporting information.
