# Supplementary material for: Status of 10 targeted genes of non‐small cell lung cancer in eastern China: A study of 884 patients based on NGS in a single institution
Source: Thorac Cancer. 2020 Jul 30;11(9):2580–9. doi: 10.1111/1759-7714.13577 (PMC7471050; doi:10.1111/1759-7714.13577)
Supplement: Supplementary file 1 — Table S1 Supporting information. [file TCA-11-2580-s001.docx]

Supplementary table 1 The detailed information of multiple mutation combinations

s

Supplementary table 2 The prevalence of each gene in surgical specimens and biopsy specimens.

|  | | No. of patients (%) | | |  |
| --- | --- | --- | --- | --- | --- |
|  | Surgical specimens | | Biopsy specimens | Total | P-value |
| *EGFR* | 433(60.6) | | 77(45.3) | 510(57.7) | **<0.001** |
| *KRAS* | 64(9.0) | | 27(15.9) | 91(10.3) | **0.008** |
| *HER2* | 24(3.4) | | 14(8.2) | 38(4.3) | **0.005** |
| *PIK3CA* | 22(3.1) | | 10(5.9) | 32(3.6) | 0.079 |
| ALK | 19(2.7) | | 2(1.2) | 21(2.4) | 0.389 |
| *BRAF* | 6(0.8) | | 4(2.4) | 10(1.1) | 0.203 |
| *ROS1* | 3(0.4) | | 2(1.2) | 5(0.6) | 0.540 |
| *RET* | 4(0.6) | | 1(0.6) | 5(0.6) | 1.000 |
| *MET* | 3(0.4) | | 1(0.6) | 4(0.5) | 1.000 |
| *NRAS* | 1(0.1) | | 0(0%) | 1(0.1) | 1.000 |
